# Supplementary material for: Quantum entanglement network enabled by a state-multiplexing quantum light source
Source: Light Sci Appl. 2025 May 12;14:189. doi: 10.1038/s41377-025-01805-1 (PMC12066727; doi:10.1038/s41377-025-01805-1)
Supplement: Supplementary file 1 — Supplementary Information [file 41377_2025_1805_MOESM1_ESM.pdf]

Supplementary Information for

Quantum entanglement network enabled by a state-multiplexing quantum light source

Yun-Ru Fan, Yue Luo, Kai Guo, Jin-Peng Wu, Hong Zeng, Guang-Wei Deng, You Wang, Hai-Zhi Song, Zhen Wang, Li-Xing You, Guang-Can Guo, and Qiang Zhou

Kai Guo, Qiang Zhou.
E-mail: guokai07203@hotmail.com, zhouqiang@uestc.edu.cn

This PDF file includes:

Supplementary text
Figs. S1 to S8
Tables S1 to S5
SI References

Supplementary Information Text

Note1. Details of silicon nitride microring resonator chip. Figure S1(a) shows the details of fiber-pigtailed Si_3N_4 microring resonator chip with thermal stability management. Figures S1(b),(c), and (d) show the measured transmission spectra of the microring from 1533 nm to 1558 nm, the resonance at the ITU channel of C46 and C34 with a quality factor of 1.29×10^6 and 1.22×10^6, respectively.

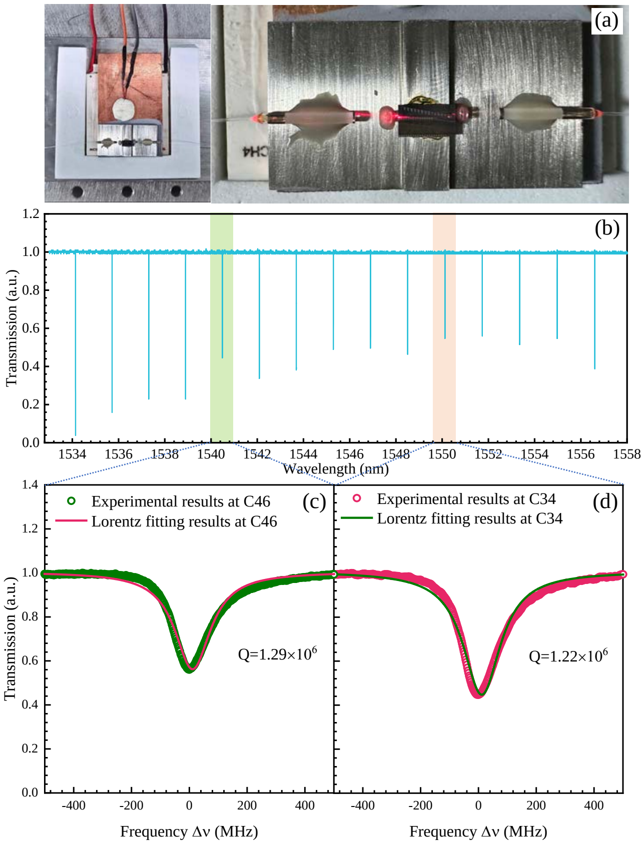

Fig. S1. Characterization of the Si_3N_4 microring chip. (a) Picture of the fiber-pigtailed Si_3N_4 device with thermal stability management. (b) Transmission spectrum from 1533 nm to 1558 nm. (c) Transmission spectrum C46 with a quality factor of 1.29×10^6. (d) Transmission spectrum C34 with a quality factor of 1.22×10^6.

Note2. Photon pairs generation with single- and dual-pump configuration. We first measure the quantum correlation property with the single-pump configuration. As shown in Fig. 2(b) in the main text, the generated photon pairs, i.e., the signal and idler photons are selected by filters and detected by superconducting nanowire single-photon detectors (SNSPDs) with a detection efficiency of 85% and dark count rate of 50 Hz. The signals of SNSPD are sent to a time-to-digital converter (TDC) to record the coincidence events. With the single pump light at the wavelength of C34, the single side count rates on the resonance of C38 are shown in Fig. S2(a). The experimental results are illustrated by the circles, and the black line is the $a{P_p}^2 + bP_p$ fitting, in which the part of $a{P_p}^2$ is the contribution of correlated photons while bP_p is the contribution of noise photons. Figure S2(b) shows the measured coincidence count rates, accidental coincidence count rates, and the ratios (CAR) between C38 and C30 with a coincidence window of 2.0 ns.

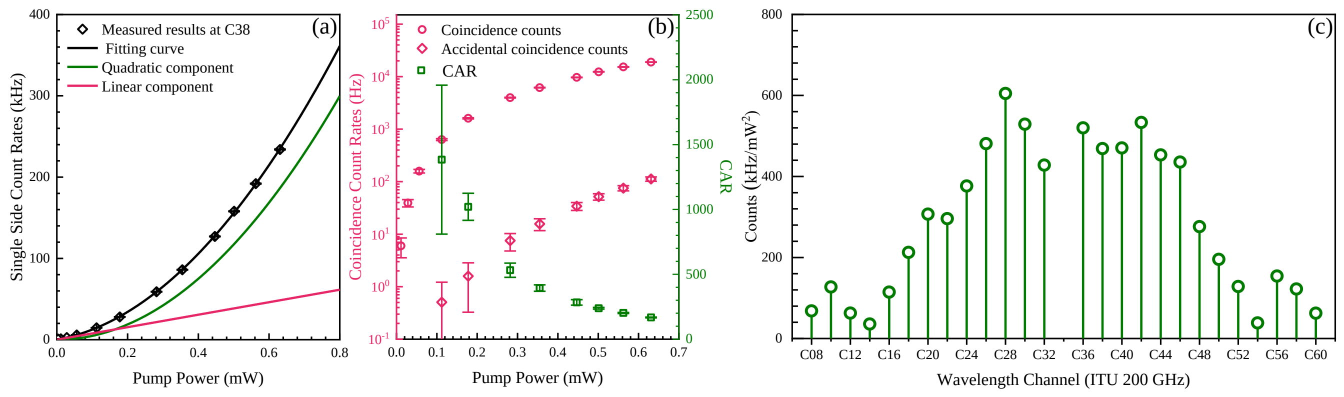

Fig. S2. Quantum correlation property of photon pairs with the single pump light at C34. (a) Single side count rates of C38 versus pump power. (b) Coincidence count rates, accidental coincidence count rates, and the coincidence-to-accidental ratio (CAR) between the photon pairs at C38 and C30 versus pump power. (c) Spectrum of the correlated photons.

To obtain the bandwidth of the spontaneous four-wave mixing process (SFWM), we further measure the single side count rates for different resonance wavelengths at different pump powers and calculate the contributions of the correlated photons by the $aP_p^2 + bP_p$ fitting as shown in Fig. S2(c). It can be seen that eight-wavelength-paired photon pairs are generated in a wavelength range of 25.6 nm, which is constrained by the phase-matching condition.

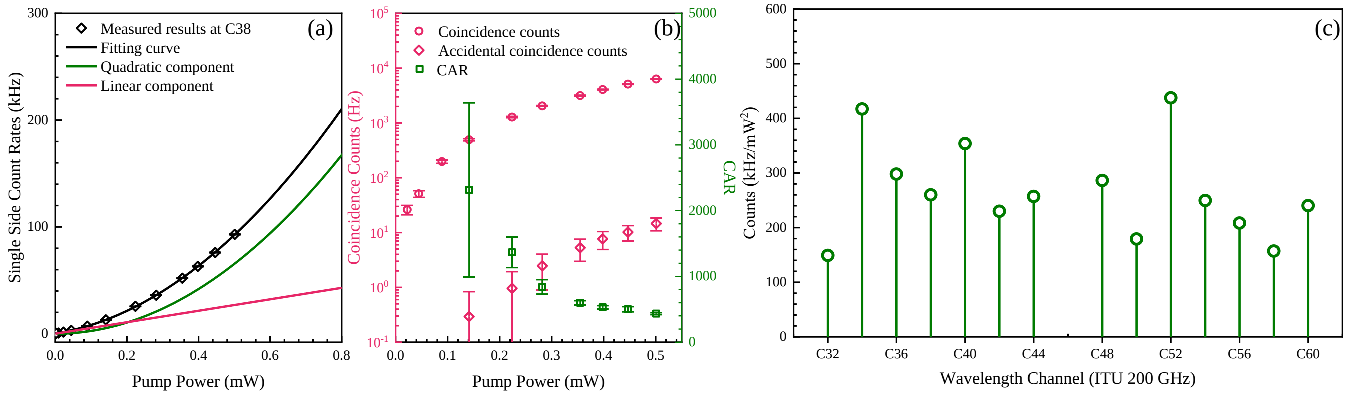

Fig. S3. Quantum correlation property of photon pairs with the single pump light at C46. (a) Single side count rates of C38 versus pump power. (b) Coincidence count rates, accidental coincidence count rates, and the CAR between the photon pairs at C38 and C54 versus pump power. (c) Spectrum of the correlated photons.

We also measure the quantum correlation property with the single pump light at the wavelength of C46 by using the same method. The single side count rates on the resonance of C38 are shown in Fig. S3(a), and the coincidence count rates, accidental coincidence count rates, and the CARs between C38 and C54 are given in Fig. S3(b). Figure S3(c) shows the contributions of correlated photon pairs at different wavelengths. Note that the other wavelengths are outside our measurements due to the lack of paired DWDMs.

To compare the properties between the non-degenerate and degenerate processes, we measure the quantum property with the dual-pump configuration, i.e., pump lights at C34 and C46 both input the Si_3N_4 chip. The single side count rates on the resonance of C38 are shown in Fig. S4(a) versus the pump powers. In our experiments, the pump power of C34 and C46 are the same. The coincidence count rates, accidental coincidence count rates, and the CARs are illustrated in Fig. S4(b). Utilizing the same approaches, the spectrum of the correlated photons is given in Fig. S4(c). It can be seen that the photon pair generation rate of the non-degenerate process is higher than that of the degenerate process, which is attributed to the

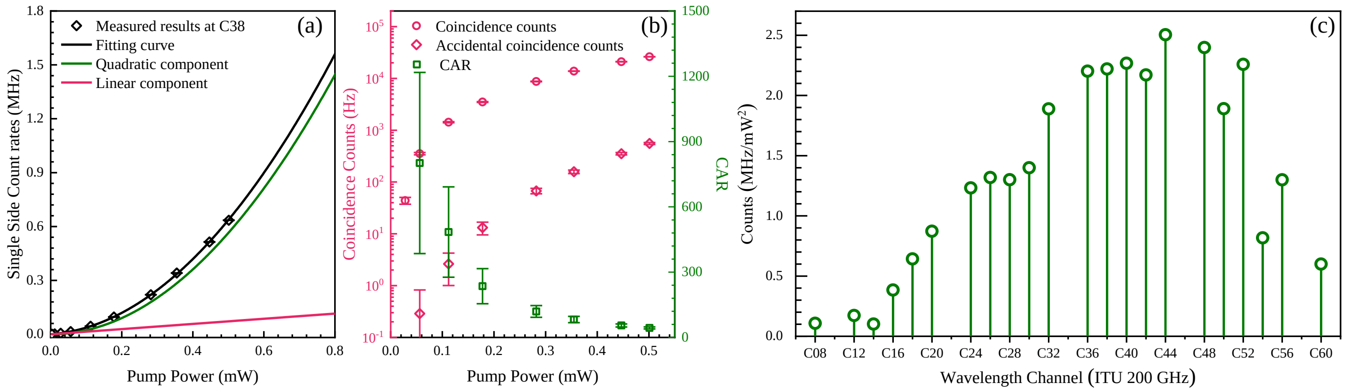

Fig. S4. Quantum correlation property of photon pairs with the dual pump lights at C34 and C46. (a) Single side count rates of C38 versus pump power. (b) Coincidence count rates, accidental coincidence count rates, and the CAR between the photon pairs at C38 and C42 versus pump power. (c) Spectrum of the correlated photons. Note that the contributions of correlated photon pairs include the non-degenerate and degenerate processes.

higher efficiency of the non-degenerate process. Besides, the non-degenerate spontaneous SFWM process is also limited by the dispersion and occurs in the same bandwidth of the degenerate process.

Furthermore, we measure the variety of CAR with the coincidence count rates by using the single- and dual-pump configuration, i.e., pump wavelength at C34, C46, and C34+C46, respectively. The results are shown in Fig. S5, which indicates lower CAR with dual-pump configuration due to the noise photons from the extra process. The Raman noise photons generated in the microring resonator need to be further reduced, and entangled two-photon states with higher generation rate could be achieved with a higher Q factor in the future.

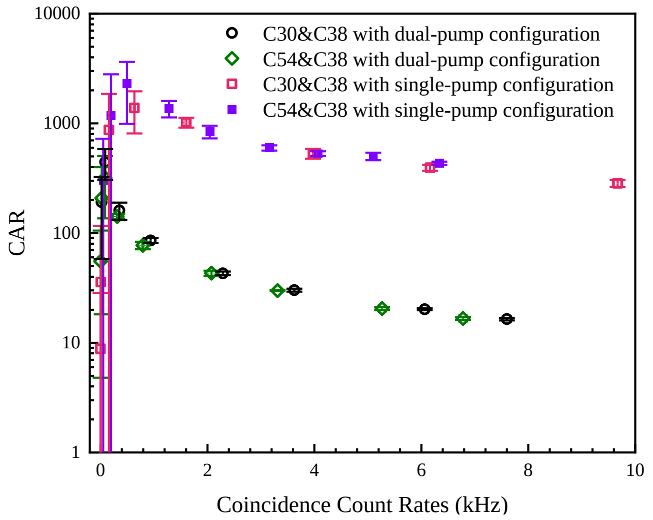

Fig. S5. Relationship between the CAR and coincidence count rates for single- and dual-pump configuration. With the dual-pump configuration, the property of photon pairs generated from the degenerate process degrades due to the photon pairs generated from the nondegenerate process are considered as “noise" and lead to more accidental coincidence events.

Note3. Quantum key distribution with single-pump configuration. We first measure the performance of a four-user fully connected quantum key distribution network with single pump light at the wavelength of C46. Six pairs of the generated entangled photons at wavelengths ranging from C34 (1550.1 nm) to C58 (1531.1 nm) are selected and input the demultiplexing/multiplexing units, as shown in Fig. S6. The losses of the photons at different wavelengths in the units are listed in Table S1. These six pairs of photons are then distributed to four users: Alice (A), Bob (B), Charlie (C), and Dave (D). Each user receives three channels/wavelengths and shares an entanglement state with every other user in the network. It is worth noting that the quantum state is encoded in the degree of freedom of energy and time, which is immune to the dispersion-induced phase instability in the quantum key distribution(1). Besides, the dispersion caused broadening of coincidence window is negligible for the generated photon pairs with a spectral width of ∼200 MHz in our experiment.

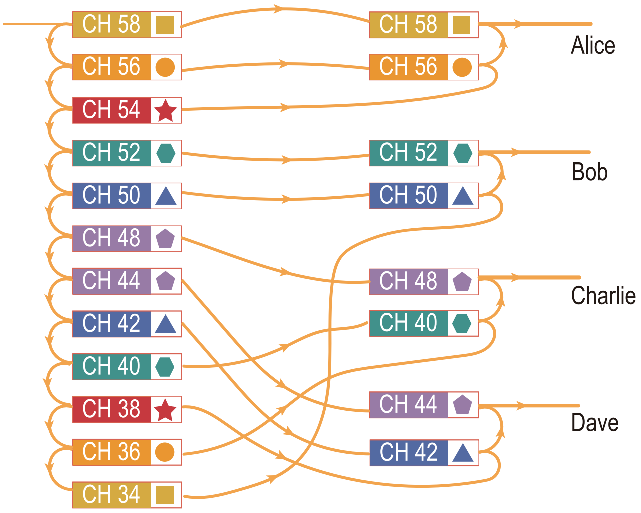

Fig. S6. Demultiplexing/multiplexing units. Six bipartite states are selected to create a fully connected network between four users.

Table S1. Losses of the demultiplexing/multiplexing unit at different wavelengths with single-pump configuration.

Users	Channel	Wavelength (nm)	Loss (dB)
Alice	C58	1531.12	2.59
	C56	1532.68	3.35
	C54	1534.25	3.27
Bob	C52	1535.82	3.26
	C50	1537.40	4.58
	C34	1550.12	5.66
Charlie	C48	1538.98	4.00
	C40	1545.32	4.99
	C36	1548.51	5.30
Dave	C44	1542.14	3.72
	C42	1543.73	5.33
	C38	1546.92	5.47

As shown in Fig. S7, we use the BBM92 protocol to analyze the property of quantum key distribution between Alice and Bob. Alice/Bob splits their photons with a 50:50 beam splitter (BS), which performs the random choice of measurement basis between Z ($0/\pi$) and X ($\frac{\pi}{2}$ / $\frac{3\pi}{2}$). It is worth noting that an attenuated continuous-wave laser is also injected into the unbalanced Michelson interferometer for phase stabilization based on feedback control. We characterize the performance of quantum key distribution between four users with six pairs of entangled photons - twelve wavelengths, as summarized in Table S2.

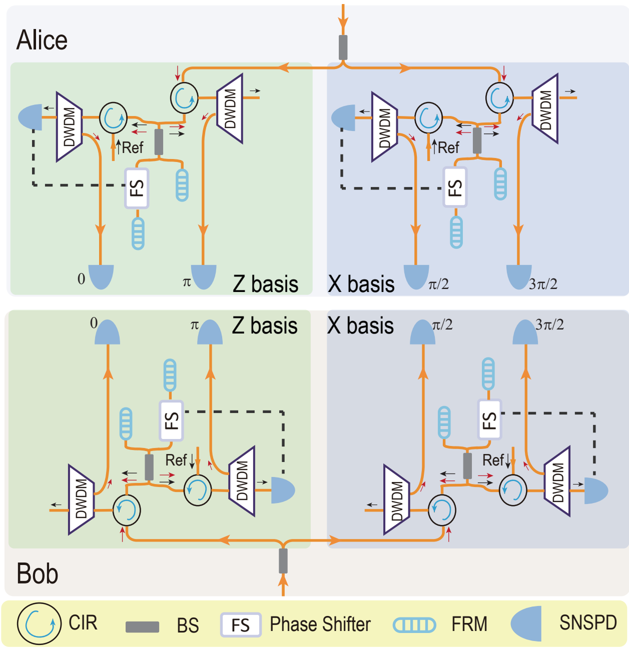

Fig. S7. Measurement setup for quantum key distribution using BBM92 protocol with feedback. The black arrows represent the direction of the reference light, and the red arrows represent the direction of the signal/idler photons.

Table S2. Measured results of quantum key distribution between four users.

Users	$N_{sift}(Hz)$	Visibility (%)	QBER (%)	SKR (bps)
A&B	336.0	93.25	3.37	178.7
A&C	280.8	88.56	5.72	85.4
A&D	283.8	90.73	4.63	114.8
B&C	337.8	92.90	3.55	173.3
B&D	298.7	86.54	6.73	65.0
C&D	333.5	92.51	3.74	164.4
Total				781.6

Note4. Losses with dual-pump scheme. With dual-pump configuration, we use eight DWDMs in the demultiplexing/multiplexing unit as shown in Fig. 2(d). The losses of each channel and each component are shown in Table S3 and Table S4, respectively.

Table S3. Losses of the demultiplexing/multiplexing unit at different wavelengths with dual-pump configuration.

Users	Channel	Wavelength (nm)	Loss (dB)
Alice	C38	1546.92	2.04
Bob	C42	1543.73	2.35
Charlie	C54	1534.25	3.19
	C50	1537.40	3.72
Dave	C26	1556.55	2.39
	C30	1553.33	3.24

Table S4. Losses of different components

Components	Loss (dB)	Efficiencies
Coupling	1.5	71%
DWDM-C46	0.8	83%
DWDM-C34	0.7	85%
PC1	0.1	98%
PC2	0.1	98%
PC3	0.2	96%
PC4	0.1	98%
SNSPD1	1.0	79%
SNSPD2	1.0	80%
SNSPD3	1.3	74%
SNSPD4	0.9	82%

Note5. Performance comparison of quantum key distribution based on the BBM92 protocol. The performance comparison of a fully connected quantum key distribution network based on BBM92 protocol with multiple users is listed in Table S5.

Table S5. Performance comparison of a fully connected quantum key distribution network based on BBM92 protocol.

Reference	Basis	Users	Wavelengths	SKR(bps)
Ref(2)	Polarization	4	12	3∼15
Ref(3)	Polarization	8	16	58∼304
Ref(4)	Energy-time	4	12	58∼252 (1298 in total)
Our work	Energy-time	4	6	22∼1230 (1946 in total)

Yun-Ru Fan, Yue Luo, Kai Guo, Jin-Peng Wu, Hong Zeng, Guang-Wei Deng, You Wang, Hai-Zhi Song, Zhen Wang, Li-Xing You, Guang-Can Guo, and Qiang Zhou

Note6. Stability of the experimental system. Figures S8(a) and (b) show the frequency stability of the two lasers, indicating that the frequency shifts are within 1 MHz. After passing through the microring resonator, the output power at the resonant wavelengths is shown in Fig. S8(c), which demonstrates the stability of our setups.

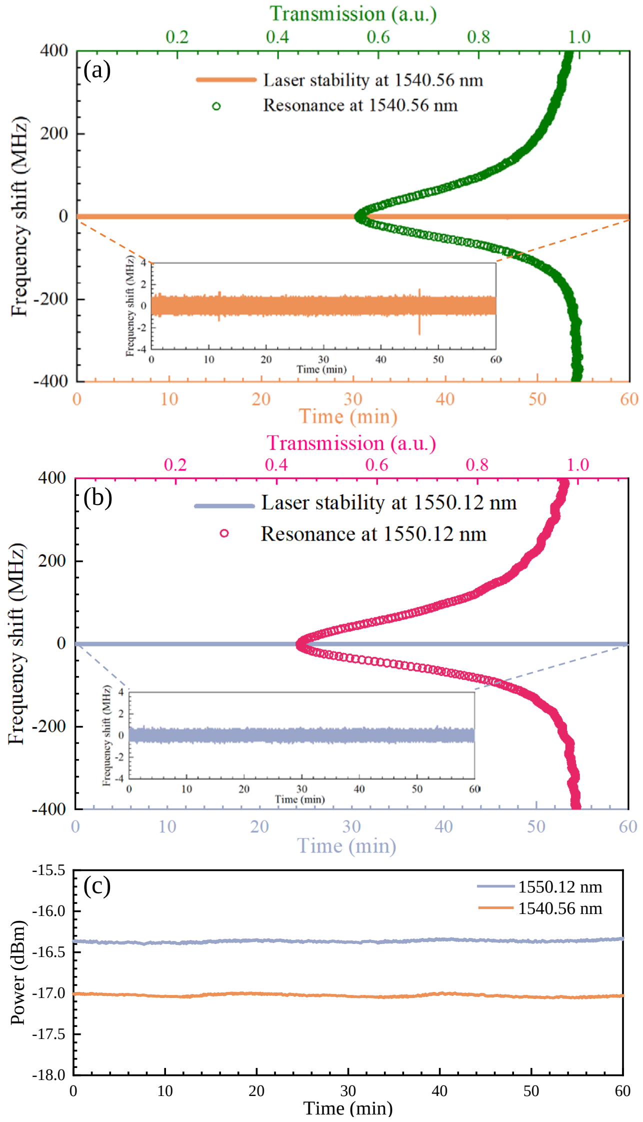

Fig. S8. Frequency shifts of pump lasers compared with transmissions of the microring resonator at (a) 1540.56 nm, and (b) 1550.12 nm, respectively. (c) Measured powers of the two pump lasers at the resonant wavelengths after passing through the microring resonator.

Note7. Theoretical analysis of SKR. The performance of a quantum light source could be characterized by coherence time or the width of the coincidence window $\Delta\tau$ and the mean photon number $\bar{n}$ per coincidence window. The coincidence rates C and the accidental coincidence count rates A can be written as

$$C = \bar{n}\frac{1}{\Delta\tau} \quad [1]$$

$$A = \bar{n}^2\frac{1}{\Delta\tau} \quad [2]$$

Coincidence-to-accidental ratio (CAR), which is used to measure the signal-to-noise ratio of the quantum light source, can be calculated by the ratio between the coincidence count rates and the accidental count rates,

$$CAR = \frac{C}{A} = \frac{1}{\bar{n}} \quad [3]$$

The visibility V of Franson interference without subtraction of accidental coincidences is limited by the number of coincidences in the interference maximum, i.e., C and the number of accidental ones, i.e., A. The visibility can be expressed as

$$V = \frac{C-A}{C+A}. \quad [4]$$

In quantum entanglement distribution networks with BBM92 protocol, the SKR can be expressed as(4–8),

$$SKR \geqslant N_{sift} \times [1 - f(\delta_b) \times H_2(\delta_b) - H_2(\delta_p)] \quad [5]$$

where N_{sift} is the sifted key rate with $N_{sift} = C + A$, $f(\delta_b)$ characterizes the error correction efficiency with respect to Shannon's noisy coding theorem, $\delta_{b,p}$ is the bit or the phase error rate respectively for the X- and Z-basis measurements, and $H_2(\delta_{b,p})$ is the binary entropy function. For the energy-time entanglement, the X- and Z-basis measurements are symmetric, resulting in $\delta_b = \delta_p = x$, where x is the overall QBER. In the experiments, x can be calculated by

$$x = QBER = \frac{A}{C+A} = \frac{1-V}{2} = \frac{1}{CAR+1}, \quad [6]$$

Therefore, $H_2(\delta_{b,p})$ can be expressed as $H_2(x) = -x\log_2 x - (1-x)\log_2(1-x)$. In our calculation, the value of $f(\delta_b)$ is set to 1.2 following the approach in the reference of (8). Then, the SKR can be expressed as

$$\begin{aligned} SKR &\geqslant N_{\text{sift}} \times [1 - 2.2H_2(x)] \\ &\geqslant (C+A) \times \{1 - 2.2[-x\log_2(x) - (1-x)\log_2(1-x)]\} \\ &\geqslant \frac{1-x}{\Delta\tau \times x^2} \times \{1 - 2.2[-x\log_2(x) - (1-x)\log_2(1-x)]\} \end{aligned} \quad [7]$$

Therefore, the SKR depends on the property of the quantum light source, i.e., the coincidence count rates and the CAR with a given coincidence window. To further improve the SKR of the entanglement-based QKD network, a quantum light source with a smaller coincidence window or coherence time can be utilized, which can be realized by utilizing dual-Mach-Zehner microring device(9) and dual-microring device with parity-time symmetry(10). Besides, with a certain quantum light source, the SKR can be optimized with CAR(11).

References

1. W Tittel, J Brendel, N Gisin, H Zbinden, Long-distance bell-type tests using energy-time entangled photons. *Phys. Rev. A* **59**, 4150 (1999).
2. S Wengerowsky, SK Joshi, F Steinlechner, H Hübel, R Ursin, An entanglement-based wavelength-multiplexed quantum communication network. *Nature* **564**, 225–228 (2018).
3. SK Joshi, et al., A trusted node–free eight-user metropolitan quantum communication network. *Sci. advances* **6**, eaba0959 (2020).
4. W Wen, et al., Realizing an entanglement-based multiuser quantum network with integrated photonics. *Phys. Rev. Appl.* **18**, 024059 (2022).
5. N Gisin, G Ribordy, W Tittel, H Zbinden, Quantum cryptography. *Rev. modern physics* **74**, 145 (2002).
6. X Ma, CHF Fung, HK Lo, Quantum key distribution with entangled photon sources. *Phys. Rev. A* **76**, 012307 (2007).
7. J Yin, et al., Satellite-to-ground entanglement-based quantum key distribution. *Phys. Rev. Lett.* **119**, 200501 (2017).
8. J Yin, et al., Entanglement-based secure quantum cryptography over 1,120 kilometres. *Nature* **582**, 501–505 (2020).
9. C Wu, et al., Bright photon-pair source based on a silicon dual-mach-zehnder microring. *Sci. China. Physics, Mech. & Astron.* **63**, 220362 (2020).
10. N Chen, et al., Parity-time-symmetry-enabled broadband quantum frequency-comb generation. *Phys. Rev. A* **110**, 023714 (2024).
11. YR Fan, et al., Entanglement networks with fiber-pigtailed silicon nitride microring in *2023 ACP/POEM.* (IEEE), pp. 1–3 (2023).
